# Insights gained from computational modeling of YAP/TAZ signaling for cellular mechanotransduction

**DOI:** 10.1038/s41540-024-00414-9

**Published:** 2024-08-15

**Authors:** Hamidreza Jafarinia, Ali Khalilimeybodi, Jorge Barrasa-Fano, Stephanie I. Fraley, Padmini Rangamani, Aurélie Carlier

**Affiliations:** 1 MERLN Institute for Technology-Inspired Regenerative Medicine, Department of Cell Biology-Inspired Tissue Engineering, Maastricht University, Maastricht, The Netherlands; 2 Department of Mechanical and Aerospace Engineering, University of California San Diego, La Jolla, CA 92093-0411 USA; 3 Department of Mechanical Engineering, Biomechanics Section, KU Leuven, Leuven, Belgium; 4 Department of Bioengineering, University of California San Diego, La Jolla, CA 92093-0411 USA

**Keywords:** Biophysics, Systems biology

## Abstract

YAP/TAZ signaling pathway is regulated by a multiplicity of feedback loops, crosstalk with other pathways, and both mechanical and biochemical stimuli. Computational modeling serves as a powerful tool to unravel how these different factors can regulate YAP/TAZ, emphasizing biophysical modeling as an indispensable tool for deciphering mechanotransduction and its regulation of cell fate. We provide a critical review of the current state-of-the-art of computational models focused on YAP/TAZ signaling.

## Introduction

Cells react to various environmental cues through a myriad of signaling pathways. Increasing experimental evidence now points to the activation of these signaling pathways not just by biochemical cues but also by mechanical cues resulting in mechanochemical regulation of cell signaling. Here, we focus on the activation of YAP (Yes-associated protein) and TAZ (transcriptional coactivator with PDZ-binding motif), known collectively as YAP/TAZ, due to the abundance of experimental data available (reviewed in refs. ^1–4^). YAP/TAZ mediates the cellular response to biomechanical stimuli, including but not limited to extrinsic ECM stiffness cues, cell–cell contact, intrinsic cytoskeletal stiffening and contractility^5^ or shear stress, and biochemical signals, such as ECM composition or soluble extracellular ligands^2,6–9^. Originally, YAP/TAZ was discovered as the effectors of the Hippo signaling pathway, which plays a crucial role in regulating tissue and organ size^10^. In recent years, however, YAP/TAZ has been found to regulate various cellular functions such as cell proliferation, differentiation, apoptosis, migration, cell lineage fate determination, and cellular circadian clocks^4,11–13^. YAP/TAZ is also involved in tissue regeneration, cell response to inflammation^14,15^ and tissue injury^16,17^, and disease pathologies such as cancer^18^. As such, YAP/TAZ is now well accepted as a fundamental readout of cellular mechanotransduction. A more comprehensive review of Hippo-mediated YAP/TAZ activity in development, homeostasis, and disease can be found here^4^.

**Fig. 1:**
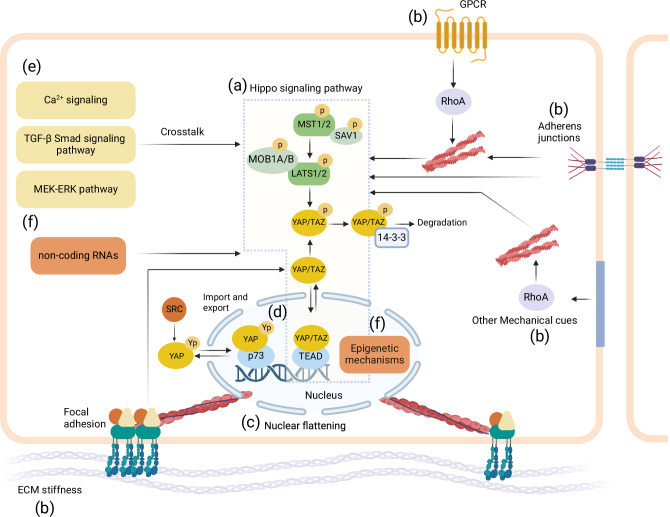
A selection of mechanisms that influence YAP/TAZ signaling, for more details we refer to refs. ^19,20^. **a** Hippo pathway, **b** upstream signaling mechanisms that may involve biomechanical cues such as ECM stiffness, shear stress, cell–cell contact, and biochemical cues such as GPCRs regulate YAP/TAZ signaling, **c** an example of a Hippo-independent pathway, **d** YAP/TAZ binding to transcription factors regulate the expression of specific target genes associated with various cellular functions, Yp refers to tyrosine phosphorylated YAP, **e** YAP/TAZ signaling crosstalk with other signaling pathways, **f** emerging mechanisms (such as epigenetic factors) influencing YAP/TAZ activity. More detailed schematics of the signaling pathways can be found in Figs. 2, 3, 4, and 5. Created with BioRender.com.

These findings have opened new questions about how the major regulator of YAP/TAZ, the Hippo signaling pathway, is integrated with other major pathways that regulate such diverse cell functions. The Hippo pathway is initiated upon phosphorylation of the MST1/2 (mammalian Ste20 kinase1/2) complex by various upstream signaling components such as focal adhesions, cell adherens junctions, and G protein-coupled receptors (GPCRs) (reviewed here^19,20^) (see Fig. 1a, b). When the Hippo signaling pathway is activated, YAP/TAZ are phosphorylated. This phosphorylated YAP/TAZ undergoes subsequent cytoplasmic sequestration and degradation^21^. Conversely, when the Hippo signaling pathway is off, unphosphorylated YAP/TAZ can enter the nucleus to regulate gene expression through association with DNA-binding transcription factors such as TEADs (TEA domain transcription factors)^22,23^. Recent studies have shown that YAP/TAZ-mediated cellular responses can involve Hippo-independent pathways as well^2,19,20,24,25^. Nuclear flattening mediated by stress fibers and lamin A function can influence YAP/TAZ signaling^9,26^, see Fig. 1c. Phosphorylation can also be a positive trigger for YAP activity. The phosphorylation of YAP at tyrosine 357 (Y357) by Src in response to mechanical stimuli^27^ or by c-Abl kinase in response to DNA damage^28^ has been associated with apoptosis^29^, see Fig. 1d. Interestingly, crosstalk between Hippo-independent and Hippo-dependent pathways have also been described. For example, the mechanical environment can modulate F-actin levels^2,30,31^, which in turn, can influence YAP/TAZ signaling through nuclear flattening^9,26^ or Hippo pathway LATS1/2 (Large tumor suppressor kinase 1/2)-dependent mechanisms^32–34^ (see Fig. 1).

In addition to this well-characterized pathway, the dynamic process of cellular signaling, such as feedback loops and crosstalk between various signaling pathways^35–38^ (see Fig. 1e), emerging role of epigenetic mechanisms^39–41^ (see figure f), and the effect of cell shape and culture dimensionality^2,24,42^ also influence YAP/TAZ signaling. The field of systems biophysics has seen a growing interest in YAP/TAZ signaling, marked by an increase in computational modeling studies since 2016. This trend reflects efforts to unravel the complex dynamics and functions of YAP/TAZ through computational approaches. Indeed, computational models offer a systematic methodology that can capture the initial conditions of a cell’s signaling state, arising from baseline experimental conditions as well as newly imposed stimuli, multiplexed stimuli, non-linear interactions, and feedback loops between various mechanical and biochemical signals. This enables models to decouple the effect of different parameters which can help to explain seemingly conflicting results, estimate the rates of biochemical reactions, and produce testable hypotheses for experimental investigation of YAP/TAZ signaling. These computational models could also assist in determining which mechanisms regulate YAP/TAZ in specific cell types or under certain physiological or pathological circumstances.

In this review, our goal is to offer the scientific community an overview of YAP/TAZ studies that use mathematical models to enhance our understanding of the complex behavior of YAP/TAZ signaling. We focus our discussion on how modeling can provide valuable insight into the different aspects of YAP/TAZ mechanotransduction, including upstream mechanical or biochemical factors, the activation of YAP/TAZ via Hippo kinases or cytoskeleton components, the dynamics of YAP/TAZ nuclear import/export, ECM-cytoskeleton-nucleus mechanical coupling, and the crosstalk of YAP/TAZ signaling with other signaling pathways. To achieve this, our approach includes explanations of methodologies, simulated pathways, model components, assumptions, key findings, and validation approaches (see Tables 1–3 for a summary of the models discussed). By summarizing the capabilities of each model (see Table 4), we anticipate that this review will be useful for the mechanotransduction community to integrate context-specific experimental readouts into comprehensive, generalizable, and predictive mathematical frameworks. Our discussion is restricted to mechanistic, white-box models, and we do not discuss data-driven techniques^43,44^.

**Table 1:** Summary of models for YAP/TAZ activation and nuclear translocation pathway initiated by upstream signaling mechanisms

			Model compartments	Model components							
				Stimuli	Integrin dynamics	FAK-RhoA activation	Cytoskel. regulation	Hippo kinases	YAP/TAZ dynamics		Code
									Act. & import	Export	
ODE	Implicit forces	Sun et al.^45^	Cytosol, Nucleus	ECM stiffness LATS	–	✓	✓	✓	Import depends on F-actin and myosin plus a baseline rate	Dependent on active LATS plus a base line rate	–
		Khalilimeybodi et al.^78^	ECM, Cytosol ER, Nucleus, Plasma, ER and nuclear membranes	ECM stiffness GPCR Ca2+ flux	–	✓	✓	✓	Adapted from^58^	Adapted from^58^	Github
ODE & PDE		Scott et al.^58^	Cytosol, Nucleus, Nuclear membrane (spatial model)	ECM stiffness	–	✓	✓	–	Activation depends on F-actin,myosin; Import depends on NPC activity; NPC activity depends on LaminA, F-actin, and myosin; LaminA activity depends on F-actin.	baseline rate	VCell
PDE		Eroume et al.^62^	Cytosol, Nucleus, Nuclear membrane (spatial model)	ECM stiffness	—	✓	✓	–	Depends on F-actin and myosin	–	VCell
Stochastic	Explicit forces	Zhang et al.^70^	Focal adhesions, Nucleus	ECM stiffness	✓	–	–	–	YAP Nucl/Cyto depends on nuclear flattening	–	zenodo
		Cheng et al.^69^	Focal adhesions, Cytosol, Nucleus	ECM stiffness	✓	✓	✓	–	Import depends on NPC diameter and YAP unfolding force, NPC diameter depends on perinuclear forces, which in turn depends on myosin and F-actin	–	–

			Main findings	Model validation	Cell type
ODE	Implicit forces	Sun et al.^45^	A novel model to convert ECM stiffness to biochemical signals; Synergetic effects of mechanosensing and Hippo pathways explained by LATS regulation of F-actin; Adhesion is a more robust regulator of YAP/TAZ than other cytoskeleton regulators; FAK overexpression rescues YAP/TAZ activity in soft environments	Sensitivity analysis of kinetic rates and comparison with the overexpression or inhibition of the actin cytoskeleton in experiments, Comparison of YAP/TAZ nuclear fraction with the experiment performed for a range of ECM stiffness.	MSC, MEC, NMuMG
		Khalilimeybodi et al.^78^	A network model including crosstalk of seven signaling pathways to elucidate the underlying mechanism behind the contradictory effects of Ca2+ on YAP/TAZ signaling;	Model predicts Ca2+ influence on YAP/TAZ under diverse conditions; Comparing time course variations of several model components with experimental data; Comparing steady-state response of adhesion and cytoskeleton components to ECM stiffness with experimental data	similar to^58^
ODE & PDE		Scott et al.^58^	Extending the ODE model of Sun et al.^45^ to incorporate the impact of cytoskeleton on active nuclear pores influencing YAP/TAZ import; Showing non-linear effect of substrate activation area (through changes in culture dimensionality) on YAP/TAZ nuclear localization; Crucial roles of cell shape and nuclear shape in determining YAP/TAZ response to substrate stiffness	Activation of key species in response to stiffness at steady-state in the compartmental ODE model	Lung fibroblast, epithelial, endothelial, brain, kidney, and bone cells
PDE		Eroume et. al^62^	YAP/TAZ nuclear translocation can be modulated by membrane binding kinetics of FAK and RhoA; Membrane-bound FAK results in a higher and more robust nuclear translocation of YAP/TAZ; Cell spreading increases YAP/TAZ nuclear translocation	Comparison the effect of diffusion and membrane localization of FAK and RhoA with experimental data	Not specified
Stochastic	Explicit forces	Zhang et al.^70^	A motor–clutch model used to show the inhibition of integrin binding rate (used to mimic HAVDI ligation) reduces nuclear deformation and YAP/TAZ Nucl/Cyto ratio.	Comparing traction forces, nuclear flattening, and YAP Nucl/Cyto ratio in response to ECM stiffness with experimental data	hMSC
		Cheng et al.^69^	Proposing a model that integrates motor–clutch dynamics of focal adhesions with the cytoskeleton regulation module of Sun et al.^45^; Developing a framework to model broader ECM mechanical behaviors including viscosity, viscoelasticity, and viscoplasticity	Comparing the dependence of YAP/TAZ Nucl/Cyto ratio on ECM stiffness with experimental data	Fibroblast cells

**Table 2:** Summary of models focusing on nuclear import and export dynamics of YAP/TAZ

		Model compartments	Key features	YAP/TAZ dynamics	Main findings	Validation	Cell type	Code
PDE	Wehling et al.^74^	Cytosol, Nucleus (spatial model)	YAP/TAZ (de)phos. and translocation models based on 1-Hippo pathway, and 2- an alternative model	Baseline rates for YAP/TAZ (de)phos. and translocation; YAP/TAZ phos. in the cytoplasm (Hippo model), and nucleus (alternative model)	A model with YAP/TAZ phos. in the nucleus is more accurate supported by the presence of LATS within the nucleus; Different (de)phos. rates for YAP and TAZ are required to predict different distribution pattern of YAP and TAZ in the experiment	Comparing YAP/TAZ spatial distribution with experimentally measured YAP/TAZ localization	Liver cancer cell	Data Software
	Ege et. al^72^	Cytosol, Nucleus, Nucleoli (spatial model)	YAP translocation and chromatin binding model for FLIP data	Baseline rates for translocation and nucleoli binding	Translocation and binding rates are determined based on the fit to experimental data	–	fibroblast	Github

**Table 3:** Summary of models exploring the crosstalk between YAP/TAZ signaling and other signaling pathways

		Model compartments	Crosstalk with YAP/TAZ signaling	Main findings	Validation	Cell type	Code
ODE	Khalilimeybodi et al.^8^	ECM, Cytosol ER, Nucleus, Plasma, ER and nuclear membranes	Ca2+ dynamics	Under different conditions increasing Ca2+ levels can both enhance or diminish YAP/TAZ activity	See Table 1	CHL1, B16-F0 breast cancer and neural stem cells	Github
	Labibi et al.^79^	Cytosol, Nucleus	TGF-β	YAP/TAZ do not influence the import and export rates of Smad; instead, they modify Smad nuclear accumulation as a retention factor and impact TGF-β receptor activity	Time course variations of Smad complexes compared with experimental data	Epithelial cell	–
	Ghomlaghi et al.^80^	Plasma membrane, Cytoplasm, Nucleus	TGF-β	Identifying regulators and molecular switches controlling YAP-Smad/p73 complexes	Time course variations of pYAP and Smad complexes compared with experimental data	Epithelial cell	Github
	Romano et al.^38^	Cytoplasm	MEK–ERK	Identifying factors that govern linear versus switching behavior in the MST2—Raf-1 network	Raf-1 and MST activation response compared with experimental data	10 cell lines	link

**Table 4:** Summary of YAP/TAZ regulatory mechanisms included in previous studies

Regulatory mechanisms of YAP/TAZ	Sun et al.^45^	Khalilimeybodi et al.^78^	Scott et al.^58^	Eroume et al.^62^	Zhang et al.^70^	Cheng et al.^69^	Wehling et al.^74^	Ege et al.^72^	Labibi et al.^79^	Ghomlaghi et al.^80^	Romano et al.^38^
Hippo kinases	✓	✓	–	–	–	–	–	–	–	–	✓
ECM stiffness converted to biochemical signals	✓	✓	✓	✓	✓	✓	–	–	–	–	–
Focal adhesion dynamics	–	–	–	–	✓	✓	–	–	–	–	–
Cytoskeleton regulation	✓	✓	✓	✓	–	✓	–	–	–	–	–
Spatial model of cytosol and nucleus	–	–	✓	✓	–	–	–	–	–	–	–
Effect of cell shape, nuclear shape and culture dimensionality	–	–	✓	–	–	–	–	–	–	–	–
Cytosol effect on the nucleus (NPC activity or nuclear shape)	–	✓	✓	–	✓	✓	–	–	–	–	–
Import and export dynamics of YAP/TAZ	–	–	–	–	–	–	✓	✓	–	–	–
Cell–cell interaction	✓	–	–	–	✓	–	–	–	–	–	–
ECM mechanical behavior (elasticity, viscosity, plasticity)	–	–	–	–	–	✓	–	–	–	–	–
Crosstalk with other signaling pathways	–	✓	–	–	–	–	–	–	✓	✓	✓

## A mathematical framework to relate ECM stiffness to YAP/TAZ activation

The mathematical model developed by Sun et al.^45^ has served as a framework in multiple studies aimed at understanding the activation of YAP/TAZ in response to ECM stiffness. To our knowledge, this is one of the first models of YAP/TAZ nuclear translocation in response to changes in ECM properties. The model includes elements from prior models of adhesion^46^, RhoGTPase^47^, and cytoskeleton dynamics^48–50^, and is extended to offer a more comprehensive view of the relationship between cell adhesion, cytoskeleton dynamics, and the regulation of YAP/TAZ by including factors such as F-actin, myosin, and LATS within a differential equation (ODE) well-mixed model. In this model, ligand density in the ECM and ECM stiffness (notably ECM Young’s modulus) is converted into a biochemical signaling cascade, starting with the activation of focal adhesion kinase (FAK) through a second-order Hill function. This formulation is consistent with earlier studies that have indicated a Hill function as a suitable model for describing the high cooperativity of the adhesion molecules^51^. The Hill equation approach was validated by comparing the experimentally measured dependence of FAK on ECM stiffness^52^.

**Fig. 2:**
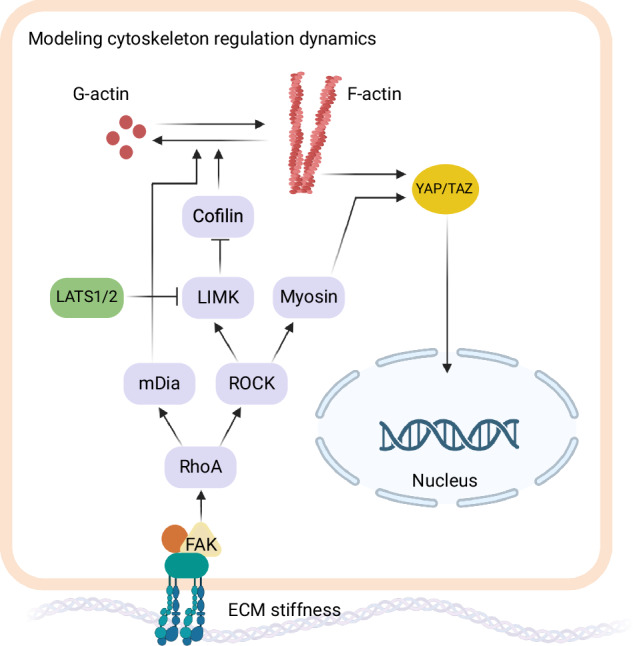
Relating ECM stiffness to YAP/TAZ activation. YAP/TAZ mechanosensing signaling pathway network suggested in ref. ^45^. Created with BioRender.com.

RhoA (Ras homolog family member A), a key GTPase, is shown to be activated downstream to FAK^53^, and upregulated with increasing ECM rigidity in 3D environments^54^. This is incorporated in the model by FAK activation leading to RhoA binding to GTP. In the model, RhoA-GTP then activates cytoskeleton regulators mDia (an actin nucleating protein in the formin family) and ROCK (Rho kinase). mDia plays a role in the polymerization of F-actin, while ROCK activates two downstream effectors: myosin and LIMK (LIM-kinase). In the model, it is assumed that the concentrations of mDia and ROCK need to surpass a threshold to initiate these downstream effects. LIMK, through phosphorylating cofilin, influences the dynamics of F-actin by inhibiting the function of cofilin in F-actin disassembly. To address the crosstalk effects of the Hippo pathway mechanosensing pathways, the interaction of LATS with LIMK-cofilin is incorporated in the model by assuming an inhibition rate for LIMK-dependent cofilin phosphorylation due to the total concentration of LATS in the cell. YAP/TAZ import to the nucleus depends on cytoskeletal mechanics which is incorporated in the model as a product of F-actin and myosin. YAP/TAZ export depends on active LATS. The model assumes baseline activation rates for RhoA, myosin, LIMK, and F-actin and a baseline YAP/TAZ translocation rate to implicitly consider the impact of additional signaling interactions. The simulated pathway is shown in Fig. 2 (see also Table 1 for the model summary).

The model produced results that were consistent with experimental observations regarding YAP/TAZ nuclear fraction at steady state by mimicking molecular interventions that either inhibit or overexpress myosin, ROCK, RhoA, F-actin, or mDia. The emphasis of model validation was on the steady states; the model did not delve into dynamic behaviors due to uncertainties in the individual kinetic parameters. YAP/TAZ nuclear accumulation in response to the ECM stiffness gave similar behavior as observed in MSC, MEC, and NMuMG cells. Additionally, variations in total FAK levels were found to determine the threshold stiffness for cell stiffness sensing, with FAK overexpression restoring YAP/TAZ activity in softer environments. Upregulation of mDia and deletion of cofilin increased YAP/TAZ nuclear translocation. The model also suggests that LATS interacts with LIMK to regulate F-actin formation, indicating a potential mechanism for the synergistic effect between the Hippo and mechanosensing pathways.

## Spatial models of YAP/TAZ signaling to unravel the impact of cell geometry, membrane-bound reactions, and substrate dimensionality

In addition to substrate stiffness, substrate dimensionality influences YAP/TAZ nuclear translocation. In two-dimensional (2D) cell culture, YAP/TAZ nuclear localization strongly correlates with substrate stiffness^2^, whereas in three-dimensional (3D) matrices, YAP/TAZ translocation can vary—increasing, decreasing, or remaining unchanged with stiffness^24,42^. Factors that spatially differ between 2D and 3D substrates, such as cell shape, cell-substrate contact area, and nuclear shape, cannot be captured by well-mixed ODE models. Capturing these features requires spatial models that can capture multicompartment reaction–diffusion equation simulations, including bulk-surface coupling to represent reactions that occur at the cell membrane^55^. Spatial modeling of cell shape and signaling pathways allows for addressing the impact of geometry, surface-volume effects, and kinase localization in signal transduction^56,57^.

**Fig. 3:**
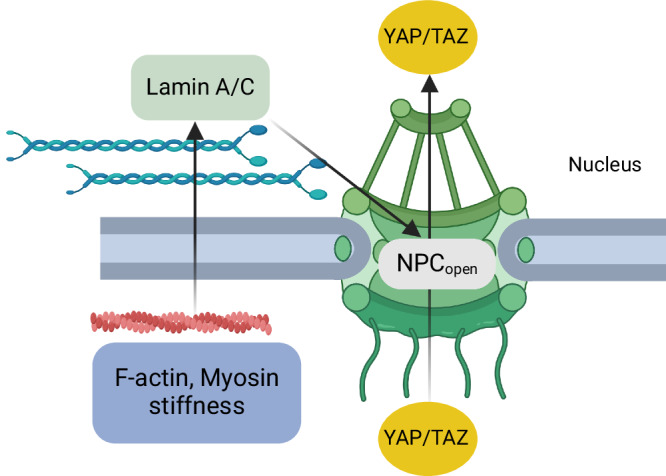
Modeling the impact of nuclear flattening on YAP/TAZ signaling. Schematic of the model used in ref. ^58^ illustrating how cytoskeletal stiffness is transferred to the nucleus via Lamin A/C, causing NPC stretching. Created with BioRender.com.

In order to systematically explore the role of cell shape and substrate dimensionality on YAP/TAZ nuclear translocation, Scott et al.^58^ extended the previous compartmental ODE model^45^ to a partial differential equation (PDE) model and included a set of reactions to reflect the mechanochemical events that affect the nuclear translocation of YAP/TAZ (see Table 1). To capture the coupling between lamin phosphorylation and cytosolic mechanical properties, the previous ODE model was extended to include a Hill equation to capture the kinetics as a function of cytosolic stiffness^45^. The cytosolic stiffness in the model, in turn, is linked to the F-actin level following a power law relationship, based on experimental observations in ref. ^59^. The authors also included stress-induced stretching of nuclear pores, which depends on F-actin, active Lamin A, and myosin. Finally, the import of YAP/TAZ was modeled to be dependent on the number of active nuclear pores (see Fig. 3). In this model, YAP/TAZ activation and translocation depend directly on the cytoskeleton regulation dynamics and indirectly on the effect of the cytoskeleton on increasing the number of active nuclear pores through Lamin A. The addition of new equations introduced a large number of free parameters in the model. This new ODE model was validated against experimental findings for FAK activation, RhoA activation, Lamin A activation, and YAP/TAZ Nuc/Cyto ratio in response to ECM stiffness in several cell types. In the case of myosin, the agreement was limited to low stiffness levels highlighting a continuing challenge of calibrating computational models to experimental data^60^.

In the subsequent step, the model was extended from a well-mixed to a 3D representation of the cell. In the spatial model, FAK activation is assumed to occur on the entire plasma membrane, and the cytoskeleton is modeled as a continuum field without resolving the actin network architecture. To test the role of substrate dimensionality (2D versus 3D conditions), the spatial model leveraged the boundary conditions. 2D culture was represented by allowing FAK activation on the boundary representing the basal side of the cell, while 3D culture was simulated by allowing the FAK activation reaction to include the entire plasma membrane. In both cases, upstream components such as pFAK, RhoA-GTP, and myosin showed a strong response to substrate stiffness in the simulation results. The response of these components to substrate dimensionality was mixed. While pFAK followed the expected trends, because RhoA had a bulk surface coupling (RhoGDP, the inactive form, was in the cytosol, but RhoAGTP, the active form, was on the membrane), the membrane area available for RhoAGTP depended on the 2D versus 3D culture conditions. As a result, the YAP/TAZ Nucl/Cyto ratio was dependent on the substrate dimensionality, particularly at higher stiffness levels. Thus, this model revealed that the interactions that take place on the plasma membrane (outside → in versus inside → out), which can differ in different substrate dimensionality conditions, can potentially explain the differences in experimental observations.

This relationship was further confirmed in simulations where the cell shape was varied by varying the radius of the basal side of the cell, while keeping the cytosolic volume and nuclear dimensions constant, essentially increasing the surface area to volume ratio of the cell. The cell shape did not significantly affect the YAP/TAZ Nucl/Cyto ratio at lower stiffness levels; however, at higher stiffness levels, elongated cells with a larger surface area to volume ratio showed higher YAP/TAZ Nucl/Cyto ratios. For a specific stiffness, elongation of the cell increased the YAP/TAZ Nucl/Cyto ratios, and this effect was more prominent in 3D. As noted above, this results from the coupling of bulk surface reactions and which surface area experiences the stimulus associated with membrane stiffness. Furthermore, for a specific cell shape, the YAP/TAZ Nucl/Cyto ratio response to stiffness is enhanced in 3D compared to 2D environments. The shape of the nucleus was also found to impact the YAP/TAZ Nuc/Cyto ratio, particularly for higher stiffness levels and larger cell sizes. Cells with elongated nuclei, indicating a larger nuclear membrane area, showed a higher YAP/TAZ Nuc/Cyto ratio because of the surface-to-volume ratio effects^61^. Interestingly, rounded cells were more robust to changes in stiffness and substrate dimensionality. The response of YAP/TAZ to substrate stiffness and dimensionality was significantly influenced by both cell shape and nuclear shape. This prediction was later validated by experimental findings where nuclear deformation was shown to be the main factor uniting the effect of mechanical cues on YAP/TAZ signaling^9^. The findings of the model suggest that increasing the surface area/volume ratio of the cell and nucleus by elongating the shape magnifies the effects of cytosolic stiffness and Lamin A activation, and this is further magnified in 3D by the activation of the full surface area, as opposed to only the basal surface area in 2D.

Additional analysis revealed that in the bulk-surface coupling, it mattered which surface area was taken into account. As noted above, reactions that depended only on the substrate presentation (outside → in) and reactions that coupled cytosolic components to the plasma membrane (inside → out) showed different dependencies on 2D and 3D stimuli. Specifically, increasing the activation area also enhanced the YAP/TAZ Cyto/Nucl ratio in relation to substrate dimensionality. Interestingly, for the 3D stimulus, the effects of increasing substrate stiffness on the YAP/TAZ Nucl/Cyto ratio showed opposite trends in the simulation under certain conditions. Increasing the stiffness led to an increase in the YAP/TAZ Nucl/Cyto ratio in two scenarios: (1) when the activation area and cell shape remained unchanged, and (2) when the cell was elongated and the activation area increased. However, increasing stiffness caused a decrease in the YAP/TAZ Nucl/Cyto ratio when the cell became more rounded. These results provided an explanation for the divergent effect of substrate stiffness in 2D and 3D environments and highlighted the important role that confining ECM conditions can play through cell and nuclear shape regulation.

Eroumé et al.^62^ also developed a similar 3D model of the cell and the nucleus based on the mechanical sensing model of Sun et al. (see Table 1) to investigate the impact of FAK and RhoA localization on YAP/TAZ nuclear translocation. Specifically, a variety of bulk-surface coupling reactions was implemented, mimicking adhesion-mediated and adhesion-independent FAK activation, and the effects of spatial localization, diffusivity, and membrane (un)binding rates on downstream YAP/TAZ signaling were explored. The results showed that scenarios where FAK is membrane-bound produce strong and enhanced YAP/TAZ nuclear translocation signals consistent with the experimentally observed cell signaling enhancement in other bulk-surface coupled signaling pathways^63–65^. For mechanosignaling specifically, the model predictions highlight the importance of adhesion-mediated versus adhesion-independent FAK activation^66^, which is dependent on the type(s) of integrins expressed and the compatibility thereof with the substrate. It was also predicted that the extent of YAP/TAZ nuclear translocation increases with cell spreading, which is related to the bulk-surface coupling. In summary, this study showed that YAP/TAZ nuclear translocation can be modulated by the bulk-surface coupling of RhoA and FAK.

## Focal adhesion dynamics and explicit mechanics can be incorporated into the YAP/TAZ signaling models via motor–clutch dynamics

**Fig. 4:**
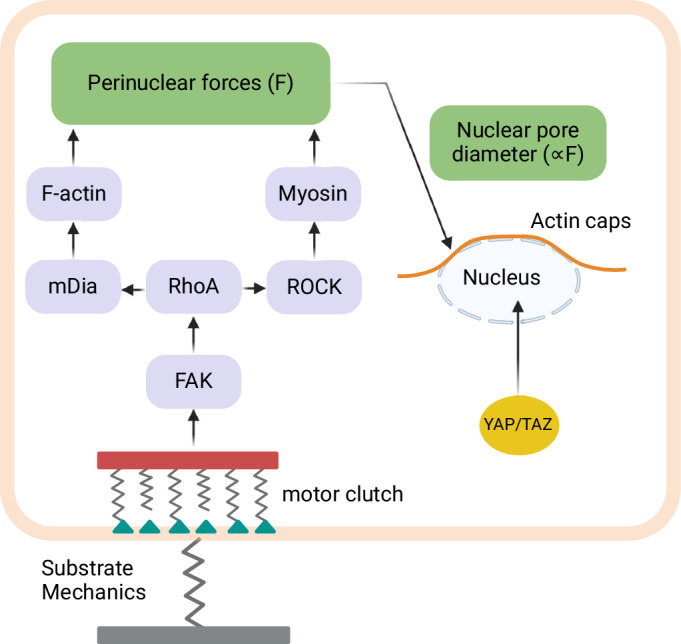
Linking matrix stiffness to YAP/TAZ activation via stochastic models. Schematic of the stochastic model integrating the motor clutch model with the YAP/TAZ signaling pathway used in ref. ^69^. Created with BioRender.com.

In earlier YAP/TAZ activation models, the signaling cascade initiates with FAK activation in response to an implicitly represented substrate, excluding the preceding steps of integrin clustering and focal adhesion dynamics, and the traction forces between the cell and the substrate. This becomes important, given experimental evidence indicating that nuclear flattening, arising from ECM–nuclear mechanical coupling plays a key role in YAP nuclear translocation^9,26^. One way to address this mechanical coupling is to explicitly include cell and matrix mechanics using motor–clutch models^67,68^ (see Fig. 4).

Cheng et al.^69^ introduced a stochastic 1D model that integrates the motor–clutch model with the ECM-FAK axis model suggested earlier^45^ (see Table 1). The model captures the ECM mechanics, focal adhesion dynamics, and integrin-FAK-myosin-YAP/TAZ signaling. This model differs from the previous models because it takes into account the density of integrins and the number of active clutch bonds. The clutch bonds are influenced by traction forces which in turn are affected by ECM featuring various mechanical properties such as elastic, viscous, viscoelastic, and viscoplastic matrices. Activation of clutch bonds triggers FAK activation, initiating the FAK-RhoA axis that ultimately leads to YAP/TAZ nuclear translocation. While the overall principle of mechanotransduction remains the same, this model adds additional detail through the clutch bonds. The model considers the import of activated YAP/TAZ into the nucleus depending on perinuclear forces similar to previous models^58^ along with constant variables representing a threshold force for YAP/TAZ unfolding^26^, and an effective stiffness for YAP/TAZ-FGNups complex. The model was validated by comparing the dependence of the YAP/TAZ Nucl/Cyto ratio on ECM stiffness with experimental data, just as the previous models did. The authors also showed that the mechanical properties of the ECM, such as short-term and long-term ECM stiffness, relaxation time, and viscosity, can influence the YAP/TAZ Nucl/Cyto ratio, opening up potential applications in designing biomaterials.

Using a simpler version of this motor–clutch model, Zhang et al.^70^ examined the impact of HAVDI peptide, a molecule used to mimic the cell–cell interactions, on the YAP/TAZ Nucl/Cyto ratio (see Table 1). In this model, explicit traction forces are considered to directly induce the deformation of a viscoelastic nucleus, resulting in nuclear flattening. A linear equation with fitting parameters was then used to capture the experimentally observed linear correlation between nuclear flattening and the YAP/TAZ Nucl/Cyto ratio^26^. Simulation results showed that modeling HAVDI ligation as a factor that inhibits the integrin binding rate in the motor–clutch model, which consequently reduces integrin clustering and nuclear deformation, explains the experimentally observed decrease in the YAP/TAZ Nucl/Cyto ratio in fibroblast cells in the presence of HAVDI.

## Mathematical models to provide insights into YAP/TAZ (de)phosphorylation and nuclear import and export dynamics

After membrane-to-cytoskeletal mechanotransduction events, YAP/TAZ can translocate to the nucleus and influence transcription. The localization, determined by the rate of nuclear import and export^26,71^, and the functionality of YAP are intricately tied to its post-transcriptional modifications, particularly phosphorylation. Recent evidence indicates that YAP undergoes dynamic shuttling between the cytoplasm and nucleus, as opposed to being stably sequestered in the cytoplasm or the nucleus^72,73^. However, the machinery regulating the dynamic YAP/TAZ import and export from the nucleus is not fully understood, although a combination of imaging techniques and mathematical modeling offers a promising approach to understanding these processes.

Ege et al.^72^ used a PDE model to recreate the experimental conditions of fluorescence loss in photobleaching (FLIP) analysis, with the aim to evaluate nuclear import and export rates of YAP as well as the rate of YAP association with TEAD in mouse and human cell lines (see Table 2). The mathematical model incorporated YAP import and export processes, along with a delta function to simulate photobleaching. Nuclear YAP was modeled either as mobile or immobile following binding to TEAD. By fitting the model to the experimental data, several variables, including association and dissociation rates with TEAD, as well as nuclear import and export rates, were estimated. The integration of quantitative photobleaching experiments and modeling revealed the significant role of nuclear export in determining the subcellular distribution of YAP.

In another study, Wehling et al.^74^ showed that the dynamic shuttling of YAP/TAZ between the cytoplasm and nucleus is dependent on the cell density in the experiment. TAZ showed considerably less dynamic behavior compared to YAP. They also observed a decoupling between YAP and TAZ localization in the nucleus and cytoplasm of liver cancer cells, with YAP showing a higher nuclear/cytoplasmic ratio at low cell density. To understand what underlies the observed behavior, a 2D PDE model, which specifically includes YAP/TAZ (de)phosphorylation and transport across the nuclear membrane, was developed (see Table 2). The authors conducted simulations under two scenarios: one representing the canonical Hippo pathway and another exploring an alternative pathway where YAP and TAZ (de)phosphorylation occurs within the nucleus and only phosphorylated YAP and TAZ are exported. Interestingly, the alternative model improved the prediction accuracy of decoupled YAP and TAZ spatial distribution patterns, providing a theoretical foundation for different (de)phosphorylation rates between the cytoplasm and nucleus. Indeed, the alternative model best matched the experimental data when YAP had a low phosphorylation/dephosphorylation ratio and TAZ had a higher phosphorylation/dephosphorylation ratio.

Note that the models discussed in this section operate under the assumption that mechanical factors do not significantly influence the observed phenomena. Consequently, these models do not explicitly capture the mechanical aspects, in contrast to the models discussed previously. Nevertheless, YAP/TAZ nuclear-shuttling dynamics are also related to physical changes in the cell, including volume fluctuations^75^. As such, extending the above models beyond biochemical factors may be an area of future modeling interest.

## Well-mixed models of YAP/TAZ signaling crosstalk with other signaling pathways to reveal molecular switches

YAP/TAZ engages in complex crosstalk with various signaling pathways such as Wnt/β-catenin signaling^35,36^, TGF-β (Transforming growth factor-beta) signaling^76^, Notch signaling pathway^37^, and MAPK signaling^77^. The interplay between these pathways contributes to the regulation of cell proliferation, differentiation, tissue homeostasis, and disease progression. Mathematical ODE models have also been used to study the regulatory mechanism in Ca2+-mediated YAP/TAZ signaling^78^ and the crosstalk of the Hippo pathway with MEK–ERK^38^ and TGF-β pathways^79,80^, see Table 3.

**Fig. 5:**
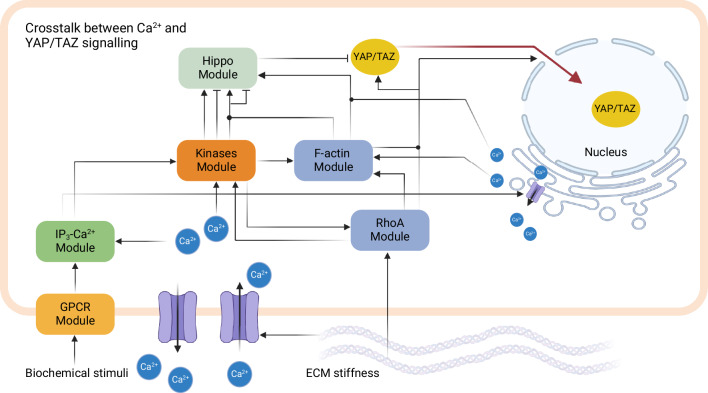
An example of mathematical modeling for crosstalk between YAP/TAZ signaling and other signaling pathways. Schematic overview of a compartmental model of Ca2+-mediated YAP/TAZ signaling. For detailed descriptions of each module, see ref. ^78^. Created with BioRender.com.

Khalilimeybodi et al.^78^ developed a compartmental ODE model that integrates multiple pathways involved in Ca2+-mediated YAP/TAZ signaling with the aim of investigating the underlying mechanism of divergent responses of YAP/TAZ to Ca2+ influx. Ca2+ levels show inconsistent YAP/TAZ responses; some studies report decreased Ca2+ influx increases YAP/TAZ activity, while others find increased Ca2+ influx activates YAP/TAZ. The distinct spatiotemporal dynamics of Ca2+ and YAP/TAZ and the crosstalk of different signaling pathways complicate understanding Ca2+-YAP/TAZ relationship through experimental methods. To address this, a network model was proposed which consists of seven interconnected modules that link biochemical and biomechanical signals to YAP/TAZ through Ca2+. These modules include the G protein-coupled receptors (GPCRs) module, which is activated upon ligand binding, the IP3-Ca2+ module, including the fluxes that regulate Ca2+ levels in the ECM, cytosol, and endoplasmic reticulum (ER), the kinases module that influences YAP/TAZ signaling downstream of Ca2+ signaling, and the RhoA, F-actin, and YAP/TAZ activation and translocation modules (see Fig. 5). The model encompasses a broad range of time scales, ranging from seconds to days, accommodating the diverse dynamics observed across different modules. The model predicted that the bistability of calcium/calmodulin-dependent protein kinase II (CaMKII) could control the transition between Ca2+-induced YAP/TAZ activation and inhibition. Additionally, examining the effect of Gq receptor stimulation frequency on YAP/TAZ activity suggests a non-linear response of YAP/TAZ to periodic GPCR stimulation, primarily influenced by LATS1/2. Through simulations conducted under diverse conditions, the model effectively captured the experimental observations that increasing Ca2+ levels can either enhance or diminish YAP/TAZ activity and the Nucl/Cyto ratio, depending on PKC (protein kinase C), DAG (diacylglycerol), and F-actin conditions through the CaMKII bistable response. These findings provide an explanation for the underlying mechanism of the controversial Ca2+-YAP/TAZ relationships and the importance of capturing initial cellular conditions when predicting responses by this pathway.

Labibi et al.^79^ and Ghomlaghi et al.^80^ used ODE models to study the complex crosstalk between the TGF-β and Hippo pathways. A crucial aspect of TGF-β signaling is the localization of transcriptional mediators, Smads, in the nucleus. When the Hippo pathway is active, YAP/TAZ primarily reside in the cytoplasm, bind to activated Smads, and hinder their nuclear accumulation, thereby attenuating TGF-β-induced transcription^81^. Under such conditions, interfering with YAP/TAZ phosphorylation can promote nuclear accumulation of both YAP/TAZ and Smads^82^. However, the role of YAP/TAZ in the nuclear localization of Smads is unclear. For the regulation of Smad nuclear accumulation two hypotheses have been proposed: one suggests different import and export rates for phosphorylated Smads^83,84^, while the other proposes nuclear retention factors have a higher affinity for phosphorylated Smads^83,85,86^. Mathematical modeling was used to reveal that YAP/TAZ does not play a role in the nuclear import of phosphorylated Smads complex. Furthermore, by integrating experimental data and ODE models, and using a Bayesian parameter estimation method, it was shown that YAP/TAZ modulate Smad nuclear accumulation by acting either directly or indirectly as a retention factor and by altering TGF-β receptor activity. These predictions generate important questions that require further experimental investigation and validation. Ghomlaghi et al.^80^ introduced another mathematical model to study the formation of YAP transcriptional complexes, specifically the shift between YAP-SMAD, responsible for cell proliferation, and YAP-p73, responsible for apoptosis. The model identified RASSF1A and ITCH as major regulators of the switch between YAP-SMAD/p73 complexes, as well as factors regulating these switches. These modeling approaches are beginning to provide quantitative prediction of cell signaling and, thereby, phenotype switches that depend on initial cellular conditions. Such models could begin to tackle questions about how functional heterogeneity arises within cellular populations.

The modeling investigation by Romano et al.^38^ of Raf-1 regulation of the MST–LATS and MEK–ERK pathways also revealed a molecular switching phenomenon. Their ODE model captures protein interactions, phosphorylation reactions, and feedback loops involving MST/LATS and ERK activation pathways. Understanding whether the MST2–Raf-1 network behaves in a linear or switch-like manner is crucial for grasping its role in cell proliferation, differentiation, apoptosis, and migration. Through mathematical modeling, the study was able to pinpoint and experimentally test the elements that determine the linear or switch-like behavior of the MST2—Raf-1 network. The model elucidated three key regulatory mechanisms that shape the network behavior: the influence of protein concentrations and affinities on the linear or switch-like behavior of the network, the impact of Raf-1 and MST2 phosphorylation on inducing switch-like behavior, and LATS1-mediated feedback phosphorylation of Raf-1. This study reveals an important mechanism by which competing protein interactions can create steep signaling switches and may extend to other instances of signaling pathway crosstalk.

## Discussion and future directions

Mathematical models provided useful insights into substrate stiffness-mediated YAP/TAZ activation combined with the effect of Hippo kinases and GPCRs^45,58,62,78^. Previous models were used to explain the divergent effect of YAP/TAZ activation in 3D environments, the effect of cell shape and nuclear shape, and the importance of cell reaction area and membrane-bound reactions in YAP/TAZ signaling^58,62^. The above mathematical models also estimated the rate of import/export of YAP/TAZ and TEAD binding dynamics^72^ and highlighted the effect of import/export rates on the distinct spatial distributions of YAP and TAZ^74^. Motor-clutch models were used to capture ECM/cytoskeleton-nuclear mechanical coupling^69,70^, aligning with experimental evidence indicating the significance of nuclear flattening and compression in YAP/TAZ signaling^9,26^. Modeling also explored the relationship between Ca2+ signaling and YAP/TAZ regulation^78^ and identified new regulatory mechanisms in TGF-β and MEK–ERK pathways^38,79,80^.

In addition, to the myriad of insights obtained, the above models also underscore the variety of methodologies employed in the modeling field. Different methodologies, capturing, for example, the spatial (PDE) or non-spatial (ODE) aspects of YAP/TAZ signaling, are chosen based on assumptions or hypotheses to abstract and simplify the multiplexed cues that the cells receive in order to gain an improved understanding (of a part) of the complex system at hand. As such, it is important to highlight the underlying assumptions and limitations. For example, models of YAP/TAZ signaling mostly study the steady-state YAP/TAZ response to ECM stiffness. These models depict the cytoskeleton, the reaction and activation areas as continuous networks with a fixed volume or area. Recent insights suggest that a more comprehensive understanding of YAP/TAZ signaling should also consider the role of the cytoskeleton and its differential engagement in 2D and 3D environments. For example, it was shown that in 3D setups, the interaction between mammary epithelial cells and the ECM leads to reduced cortical actin tension (with respect to 2D), which affects cellular mechanics and protein secretion^87^. This contrasts sharply with 2D environments, where high cortical actin tension can result in cellular stress responses, including disrupted calcium homeostasis and reduced cell viability^87^. Incorporating dynamic changes in cell and nuclear shape during cell spreading, along with discrete focal adhesions and physiologically relevant reaction areas, could improve the predictive capabilities of these models. These enhancements may capture the dynamic nuclear localization of YAP, the fast oscillations of YAP nucleocytoplasmic localization, and YAP fluctuations in motile cells^9,75^. However, achieving these improvements necessitates additional time-series data on spatial and temporal YAP localization for model calibration.

Moreover, including the effect of surface topographies^88^, investigating the potential feedback of cell contractility on ECM properties^89^, along with cell–ECM deposition^90^, and the feedback effect of YAP/TAZ activation on focal adhesions through expression of genes encoding for focal adhesion proteins^6^ may offer additional insights into the system. Differentiating between serine phosphorylation of YAP linked to proliferation and tyrosine phosphorylation of YAP by SRC and c-Abl kinases, associated with apoptosis^27,28^, could broaden the applicability of current mathematical models.

The effect of nuclear shape and nuclear compression have been suggested to unite the effect of mechanical cues on YAP/TAZ signaling^9^. Combining the motor–clutch dynamics with the YAP/TAZ signaling pathway has introduced a way to include the focal adhesion dynamics and ECM-cytoskeleton-nuclear mechanical coupling with explicit mechanics^67–69,91,92^. However, these models typically focus on singular adhesions or imply the presence of the cytoskeleton without fully accounting for the force balance within the entire cell. Integrating these existing models into a more comprehensive whole-cell model with explicit cellular mechanics, similar to^93^, would be a significant advancement. This approach would provide a more holistic understanding of how mechanical cues impact YAP/TAZ signaling at the cellular level.

To further enhance the applicability of motor–clutch models in elucidating YAP/TAZ signaling pathways, future work should focus on exploring the capabilities of these models to predict the stochastic activation patterns of YAP/TAZ, measured at the single-cell level. This exploration should involve comparisons between the stochastic predictions of motor clutch models and data from single-cell studies. Such an analysis will serve as a critical test of the model capability to reflect cellular responses to mechanical cues. Furthermore, given the complex nature of YAP/TAZ activation, which is likely influenced by multiple stochastic factors, future research should also aim to delineate the specific contributions of mechanics-induced stochasticity.

The motor–clutch framework was also used to simulate the effect of cell–cell contact^70^. However, the regulation of YAP/TAZ in multicellular environments remains less explored in mathematical studies. Signaling at adherens junctions can exert dual effects on YAP/TAZ activation, with negative regulation at high cell densities and positive regulation at low cell densities^20^. To capture the complexity of in vivo systems and understand the combined (cooperative or antagonistic) effects of cell–ECM and cell–cell interactions on YAP/TAZ regulation at physiologically or disease-relevant cell densities, it is important to incorporate YAP/TAZ regulation by, amongst others, adherens junctions^94^. Modeling multiple mechanical cells and exploring the role of cadherins could also be beneficial for a better understanding of the overall effect of substrate dimensionality and spatiotemporal YAP/TAZ dynamics^20,90,94,95^. For this purpose including the effect of cadherins into the current spatial models or extending the motor–clutch model for YAP/TAZ signaling to 2D or 3D would significantly enhance our understanding of mechanical homeostasis. Incorporating experimental data, particularly from techniques like TFM and FRET sensors, into these computational models holds great promise for advancing our understanding of the role of forces in this signaling pathway.

## How experimental data can enhance mathematical models with explicit forces

Moving towards comprehensive models with explicit mechanics necessitates a deep, quantitative understanding of forces at cell–matrix or cell–cell interfaces. Quantifying cell-generated forces represents a challenging task, but there are multiple experimental techniques that can be used to incorporate such information into YAP/TAZ mathematical models.

Traction force microscopy (TFM) is a widely used methodology to quantify the forces that cells exert on the matrix. Zhang et al.^70^ explicitly included cell traction forces in the model and used TFM to correlate them with nuclear deformation and consequent nuclear YAP re-localization. By incorporating experimentally validated explicit traction forces, their model offered a more mechanistic understanding of YAP/TAZ regulation. Another example where TFM helped in unraveling YAP/TAZ mechanisms is the work of Link et al.^96^. In this study, they revealed that combined knockdown of YAP and TAZ was more effective in reducing contractile function compared to targeting individual cytoskeletal genes, hinting at a multi-gene regulatory program essential to cell contractile functions governed by YAP/TAZ. Such studies highlight that TFM offers valuable data for validating and refining mathematical models of YAP/TAZ. Moreover, TFM can be useful for mathematical models of diverse nature. The TFM field is quickly evolving from simplified 2D systems to 3D systems of single cells or multicellular structures^97–100^, incorporating matrix viscoelasticity^101^ or matrix degradation^102^, and becoming easily accessible by biologists^103,104^.

Quantifying cell–cell force information can be challenging, particularly when studying living (embryonic) tissues. Porazinski et al.^105^ used laser ablation experiments to study tissue tension in the medaka embryos and found that the recoil velocity of the cortex in response to the cut opening was significantly reduced in YAP/TAZ knockdown embryos compared to control embryos, suggesting reduced actomyosin network tension. Such laser ablation experiments do not provide quantitative information on the cell–cell forces, but it can be used as a relative readout to calibrate mathematical models. More quantitative techniques, such as droplet-based sensors, have also been used in the context of embryonic development^106^. Other techniques have been developed for in vitro systems. Monolayer Stress Microscopy (MSM), which uses the tractions quantified by TFM to obtain tension at cell junctions through Newton’s laws, has been used to quantify intracellular and intercellular tension in cell collectives^107,108^. By including information from MSM in a mathematical model of YAP/TAZ signaling, researchers could gain insight into how changes in cell–cell forces influence YAP/TAZ activation and downstream transcriptional responses. The use of Fluorescence Resonance Energy Transfer (FRET) sensors to access cell–cell, and cell–ECM forces is a burgeoning area of interest in the mechanobiology field^109^. Min et al. used FRET technology to monitor material shrinkage or stretching. Magnetic nanocoils linked to FRET sensors revealed that nanostretching of the material leads to YAP/TAZ nuclear translocation^110^. Borghi et al. used FRET-based molecular tension sensors to measure pN-range forces transmitted between the transmembrane domain and the catenin-binding domain of E-cadherin^111^. This allowed the researchers to directly measure the tension exerted by the actomyosin cytoskeleton on E-cadherin. Price et al. used FRET-based tension sensors to measure the forces experienced by desmoplakin, a protein that links the desmosomal plaque to intermediate filaments at cell–cell interfaces^112^. FRET-based experimental data can offer insights into the intricate mechanosensing mechanisms governing YAP/TAZ activity, allowing computational models to be enriched with additional details and more realistic representations of the underlying biology.

## Going beyond physiological mechanosignaling

Many studies identified the pathological roles of the Hippo pathway, notably the importance of YAP and TAZ in cancer progression, and Piccolo et al. have recently reviewed these findings^113^. However, a growing number of studies are also showing that YAP/TAZ can exert tumor suppressor functions by its activity in peritumoral tissue and in certain tumor contexts, which Franklin et al. reviews^114^. This echoes the ability of YAP/TAZ to drive apoptosis through association with p73. In some cancer types, like ER+ breast cancer, partial mechanisms that explain this tumor-suppressor function have been identified^115–117^. Nonetheless, opportunities are ripe for modeling efforts to explain the key physical and/or molecular mediators of these two disparate outcomes in other cancer types, which could support the definitions of tumor/patient contexts and identify specific treatment strategies. For these models to be successful, genomic alterations that affect YAP/TAZ directly or signaling pathways that cross-talk with YAP/TAZ will need to be considered.

## Emerging regulatory mechanisms

Given recent studies that indicate YAP/TAZ association with chromatin-remodeling complex proteins to change chromatin structure, thus influencing accessibility and activity of target genes, the next paragraphs discuss the emerging role of the epigenetic mechanisms in regulating YAP/TAZ activity. Comprehensive reviews on this topic can be found here^39–41,118^.

It has been shown that histone modifications directly affect the transcriptional activity of YAP/TAZ by modifying chromatin structure around target genes. Histone acetylation generally enhances transcription by loosening chromatin structure, thereby facilitating access to transcriptional machinery. YAP/TAZ recruit histone acetyltransferases (HATs) to enhance transcription at specific enhancers^119–121^. Conversely, the transcription repression by YAP/TAZ is associated with the recruitment of Nucleosome Remodeling and Deacetylase (NuRD) repressor complex. This complex has both ATP-dependent chromatin remodeling and histone deacetylase (HDAC) activity to induce the compaction of nucleosomes and restrict genomic accessibility^122,123^.

Histone methylation also plays a role in modulating transcriptional outcomes, acting as either an activator or repressor of gene expression depending on the specific histone residues involved. Particularly, methylation of histone H3 is a general mechanism associated with changes in chromatin structure that influence transcriptional activity^124^. Methylation patterns are regulated by a variety of histone methyltransferases (HMTs)^125,126^. Particularly, the nuclear receptor coactivator 6 (Ncoa6), a component of the Trithorax-related MLL2/3 HMT complex, has been identified as an important mediator of Hippo pathway transcriptional responses^127^. It has also been shown that transient downregulation of YAP contributes to DNA methylation remodeling during cell differentiation, highlighting the role of YAP in epigenetic dynamics^128^.

Recent discoveries have also found a substantial impact of non-coding RNAs (ncRNAs), including microRNAs (miRNAs), long non-coding RNAs (lncRNAs), and circular RNAs on the regulation of YAP/TAZ signaling pathways. These ncRNAs are integral to both the modulation of YAP/TAZ activity and the broader regulatory networks in which YAP/TAZ participate, particularly in cancer progression^118^.

More specifically, microRNAs play multifaceted roles in regulating YAP/TAZ activities. They can directly target YAP/TAZ mRNA, leading to translational inhibition or mRNA degradation^129^. They can also affect the stability or nuclear translocation of YAP/TAZ proteins^130,131^. Moreover, miRNAs can influence the transcription of YAP/TAZ by interacting with transcription factors or core components of the Hippo signaling pathway^132^. Importantly, YAP/TAZ also regulate the expression of specific miRNAs, thus participating in intricate feedback loops^133,134^.

lncRNAs are increasingly recognized for their role in modulating YAP/TAZ. They bind to miRNA, thus affecting the expression of YAP/TAZ and Hippo pathway components^135,136^. LncRNAs can also directly interact with proteins to affect DNA transcription, RNA stability, and chromatin architecture, impacting the regulation of YAP/TAZ at multiple levels^137–139^. Additionally, lncRNAs have been found to drive aberrant liquid-liquid phase separation, influencing YAP/TAZ activity^140^. YAP/TAZ themselves can regulate the expression of certain lncRNAs, which further complicates the feedback and regulatory loops involving these molecules^141^. Similar to lncRNAs, circRNAs target miRNA, thereby modulating the expression of YAP/TAZ^142^. They also affect protein-mRNA interactions and have been reported to encode small peptides that could participate in the regulation of YAP/TAZ signaling^143,144^.

**Fig. 6:**
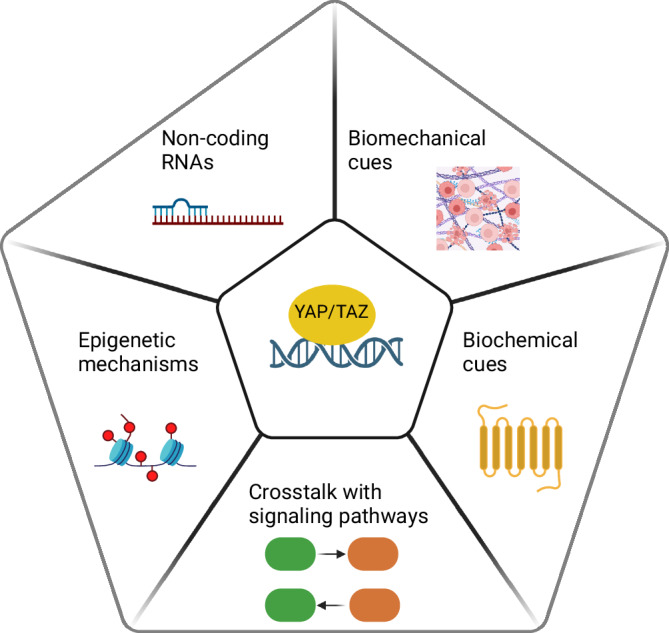
Key factors affecting YAP/TAZ signaling. This figure provides a comprehensive overview of the primary factors that affect YAP/TAZ signaling. Created with BioRender.com.

As such, to help the field move forward, future modeling efforts need to integrate the emerging epigenetic mechanisms into mathematical models of YAP/TAZ signaling. Such comprehensive models would allow us to better capture the complexities of YAP/TAZ regulation, summarized schematically in Fig. 6.

## Conclusion

The iterative process of model validation and refinement through experimental data integration has provided valuable insights into the mechanosensing mechanisms governing YAP/TAZ activity, shedding light on its complex regulatory network. Recent models highlight several emerging themes of YAP/TAZ signaling, including its physical context dependence, initial condition dependence, and mechanisms of switch-like behavior that mediate the crosstalk of YAP/TAZ with other signaling pathways. These works demonstrate the increasingly important role of mathematical models in consolidating disparate findings into generalizable frameworks that can predict diverse outcomes.
